# ADVERSE CHILDHOOD EXPERIENCES AND DEPRESSION: AGING PERCEPTIONS’ MEDIATING ROLE IN OLDER KOREAN IMMIGRANTS

**DOI:** 10.1093/geroni/igae098.4110

**Published:** 2024-12-31

**Authors:** Hajin Lee, Juyoung Park, Yuri Jang

**Affiliations:** Ewha Womans University, Seoul, Republic of Korea; University of Southern California, Los Angeles, California, United States; University of Southern California, Los Angeles, California, United States

## Abstract

In response to the sustained mental health impact of Adverse Childhood Experiences (ACEs) in later life and the heightened vulnerability of older adults in socially disadvantaged positions, we examined how early life adversities shape self-perceptions of aging and mental health among low-income older Korean immigrants. We conceptualized self-perceptions of aging as a mediator and hypothesized that the impact of ACEs on depressive symptoms would be mitigated by negatively internalized perceptions of aging. Using data from 315 older Korean residents in subsidized housing in the Los Angeles area (Mean age = 79.4), we found supportive evidence for our hypotheses. In addition to the direct effects of ACEs (B [SE] =.80 [.18], p <.001) and self-perceptions of aging (B [SE] = −.68 [.16], p <.001) on depressive symptoms, the indirect effect of ACEs on depressive symptoms through self-perceptions of aging was found to be significant (B [SE] =.12 [.05], bias-corrected 95% bootstrap confidence interval = 0.02, 0.22). Those with greater ACE exposures were more likely to hold negative perceptions of aging, which in turn led to greater symptoms of depression. Our findings elucidate a potential mechanism through which early life adversities manifest the impact on the current mental health state of low-income older Korean immigrants and highlight the importance of culturally sensitive community-based interventions to foster resilience and positive views on aging for the protection and promotion of community mental health.

